# Association of grip strength, sleep duration, and comorbidities with depressive symptoms in middle-aged and older patients with chronic lung diseases: a cross-sectional network analysis based on CHARLS data

**DOI:** 10.3389/fpsyg.2024.1472766

**Published:** 2024-11-27

**Authors:** Lu Li, Jiaqi Meng, Yinxiang Wu, Xiaoyuan Bu, Liping Gao, Zhiwei Xiao, Jiquan Chen

**Affiliations:** Department of Pulmonary and Critical Care Medicine, Third Affiliated Hospital of Naval Medical University, Shanghai, China

**Keywords:** chronic lung disease, depressive symptoms, grip strength, lifestyle, comorbidities, CHARLS

## Abstract

**Background:**

Depressive symptoms are prevalent among patients with chronic lung diseases (CLDs) and adversely impact their quality of life. This study aims to explore the association of grip strength (GS), sleep duration, and comorbidities with depressive symptoms in patients with CLDs, with an in-depth analysis of the underlying mechanisms.

**Methods:**

Based on data from the China Health and Retirement Longitudinal Study (CHARLS), this study included participants aged 45 and above with diagnosed CLDs. Depressive symptoms were assessed using the 10-item Center for Epidemiologic Studies Depression Scale (CESD-10). Binary logistic regression, subgroup analysis, and network analysis were conducted to examine the intricate relationships between GS, sleep duration, comorbidities, and specific depressive symptoms.

**Results:**

Among the 1,427 participants, 39.8% exhibited depressive symptoms. Adjusted analyses revealed that GS (OR = 0.964, *p <* 0.001) and sleep duration (OR = 0.808, *p <* 0.001) were negatively associated with depressive symptoms, whereas the number of comorbid chronic diseases (OR = 1.189, *p <* 0.001) showed a significant positive correlation with depressive symptoms. Subgroup analyses demonstrated variations in these relationships across different demographic characteristics and lifestyle factors. Network analysis pinpointed “depressed” as the core symptom, with “annoyed,” “exhausted,” and “lonely” as secondary core symptoms. The robust associations between specific factors and depressive symptoms (GS with “annoyed,” sleep and comorbid chronic diseases with “depressed”) suggested potential targets for interventions.

**Conclusion:**

This study underscores the complex interplay of GS, sleep duration, and comorbidities with depressive symptoms in patients with CLDs. These findings offer new perspectives for improving the mental health of this vulnerable population.

## 1 Introduction

Chronic lung diseases (CLDs), especially chronic obstructive pulmonary disease (COPD), are significant contributors to the rising global morbidity and mortality rates (GBD 2021 Diseases and Injuries Collaborators, 2024; GBD Chronic Respiratory Disease Collaborators, 2020; GBD 2019 Chronic Respiratory Diseases Collaborators, 2023; Adeloye et al., 2022; Al Wachami et al., 2024). With the aging of populations and increasing environmental pollution, COPD has become the third leading cause of death worldwide (GBD 2021 Diseases and Injuries Collaborators, 2024; GBD Chronic Respiratory Disease Collaborators, 2020; GBD 2019 Chronic Respiratory Diseases Collaborators, 2023; Adeloye et al., 2022; Al Wachami et al., 2024). CLDs not only impair respiratory function and quality of life but are also frequently accompanied by multiple comorbidities, with depressive symptoms being particularly common and severe (Zhang et al., 2011; Zheng et al., 2022; Yohannes and Alexopoulos, 2014). These comorbid conditions exacerbate the overall disease burden and contribute to heightened hospitalization rates, prolonged hospital stays, escalated healthcare costs, and increased mortality (Zhang et al., 2011; Zheng et al., 2022; Yohannes and Alexopoulos, 2014). Despite the well-recognized high prevalence of depressive symptoms among patients with CLDs, the contributing factors and underlying mechanisms remain insufficiently studied. Current research has focused on factors such as disease severity (Kim et al., 2014), respiratory difficulties (Bestall et al., 1999), diminished quality of life (Pedrozo-Pupo et al., 2021), and insufficient social support (Stoustrup et al., 2024). However, there is a relative paucity of research examining the relationships between potentially modifiable factors, such as grip strength (GS), sleep quality, and comorbidities, and depressive symptoms. This gap underscores the need for further investigation into these potentially alterable influences to better understand and manage depression in the context of CLDs.

GS, as a simple, quick, and cost-effective measure, is widely recognized as a reliable indicator for overall muscle strength (Norman et al., 2011). In recent years, the association between GS and various health outcomes has garnered significant attention from researchers. Evidence suggests associations between GS and cognitive function, physical performance, and metabolic health, indicating that GS may reflect not only physical but also mental resilience (Muhammad and Maurya, 2022). Additionally, lower GS has been linked to inflammatory processes, which are known contributors to depression (Granic et al., 2017). Given the prevalent decline in muscle function and overall physical capacity among patients with CLDs, an investigation into the relationship between GS and depressive symptoms could offer a novel perspective. This approach may facilitate the early detection and proactive management of high-risk individuals.

Lifestyle factors, including smoking, alcohol consumption, physical activity intensity, and sleep quality, have been demonstrated to correlate with mental health in the general population (Zhong et al., 2019). Notably, sleep disorders, in particular, are a common complication among patients with CLDs (Du et al., 2023) and a significant factor affecting quality of life. The influence of sleep duration on the development and exacerbation of depressive symptoms in patients with CLDs, however, remains uncharacterized. Given the propensity for respiratory difficulties in patients with CLDs to compromise sleep quality, a focused study on the impacts of sleep duration on their mental health holds considerable clinical relevance. Moreover, patients with CLDs frequently suffer from various comorbidities, such as cardiovascular diseases, diabetes, and osteoporosis (Cavaillès et al., 2013). These comorbid conditions not only complicate medical management but may also have profound implications for their mental health (Negewo et al., 2015). Despite its importance, research exploring the link between comorbidities and depressive symptoms in patients with CLDs remains limited. Understanding the impact of comorbidities on depression risk is essential for developing comprehensive management strategies and improving patient outcomes.

In recent years, network analysis methods have witnessed widespread application in psychiatric and psychological research (Borsboom and Cramer, 2013; Borsboom, 2017; Zhong et al., 2023). This approach can dissect the complex interactions between symptoms beyond the reach of conventional statistical techniques. Despite their potential, network analysis methods have been scarcely applied in the study of depressive symptoms among patients with CLDs. This emerging method, which explores the complex network of relationships between depressive symptoms, GS, sleep duration, and comorbidities, may provide new insights into the mechanisms of depression development and the formulation of targeted intervention strategies.

Given the current research landscape, this study aimed to address existing gaps by thoroughly exploring the associations of GS, sleep duration, and comorbidities with depressive symptoms in patients with CLDs utilizing data from the China Health and Retirement Longitudinal Study (CHARLS) (Zhang et al., 2023; Zhao et al., 2014). The specific objectives of this study are to evaluate the prevalence of depressive symptoms among patients with CLDs and their relationships with demographic characteristics, to investigate the associations of GS, sleep duration, and comorbidities with depressive symptoms, and to identify variations in these associations across different subpopulations through subgroup analyses. Additionally, the study intended to examine the complex network relationships between these factors and specific depressive symptoms using heatmaps and network analysis methods. The insights gained from this study may enhance the understanding of the influencing factors and underlying mechanisms of depressive symptoms in patients with CLDs. This study could potentially inform the development of targeted prevention and intervention strategies, thereby contributing to improvements in the mental health and quality of life of this vulnerable population.

## 2 Methods

### 2.1 Study design and data acquisition

This study was conducted using a cross-sectional design, based on the data from the 2015 iteration of the CHARLS database (Zhao et al., 2014). CHARLS is a national, long-term survey project designed to gather detailed health and socioeconomic data from individuals aged 45 and above in China. The was implemented through a multi-stage stratified probability sampling method, covering 150 counties and 450 villages/communities across 28 provinces in China, thereby ensuring a robust representation of the national demographic.

**Figure 1:**
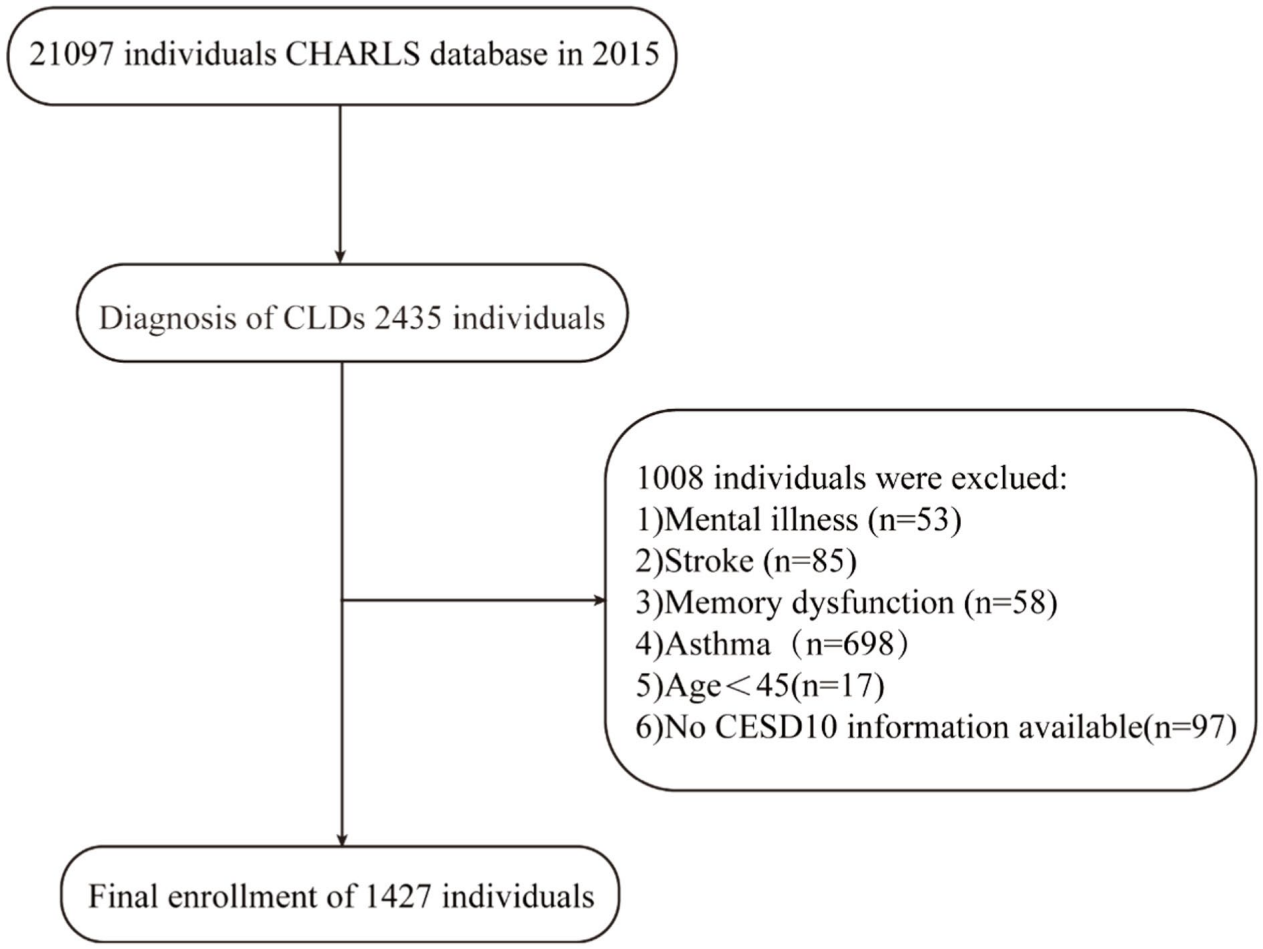
Flowchart of participant selection. CLD, chronic lung disease; CHARLS, China Health and Retirement Longitudinal Study; CESD-10, 10-item Center for Epidemiologic Studies Depression Scale.

The target population for this study was comprised of individuals aged 45 years and older with CLDs. Participants were selected based on their age and self-reported diagnosis. The study specifically excluded individuals with asthma, stroke, psychiatric disorders, memory-related diseases, or incomplete CESD-10 data. Based on these criteria, the recruitment process culminated in the inclusion of 1,427 participants, with the detailed selection procedure illustrated in Figure 1.

Ethical approval for the CHARLS study was obtained from the Biomedical Ethics Committee of Peking University (IRB00001052-11015). This study utilized publicly available anonymized data, thus no additional ethical approval was required.

### 2.2 Measurement of variables

#### 2.2.1 Primary outcome variable: depressive symptoms

The evaluation of depressive symptoms was conducted using the 10-item Center for Epidemiologic Studies Depression Scale (CESD-10) (Zhu et al., 2024). The CESD-10 includes 10 items, with each item scored from 0 to 3, allowing for a total score ranging from 0 to 30. Following established research protocols, a total CESD-10 score of 10 or higher was considered indicative of depressive symptoms. Moreover, nine specific depressive symptoms were identified based on the individual CESD-10 items. as detailed in Table 1. The internal consistency of the CESD-10 scale was assessed using Cronbach’s alpha coefficient, which indicated an acceptable level of reliability for evaluating depressive symptoms among participants in this study.

**Table 1:** CESD-10 item descriptions.

Code	Term	Description	Note
C1	I was bothered by things that do not usually bother me.	Annoyed	–
C2	I had trouble keeping my mind on what I was doing.	Distracted	–
C3	I felt depressed.	Depressed	–
C4	I felt everything I did was an effort.	Exhausted	–
C5	I felt hopeful about the future.	Hopeless	Reverse scored
C6	I felt fearful.	Fearful	–
C7	I was happy.	Unhappy	Reverse scored
C8	I felt lonely.	Lonely	–
C9	I could not get “going.”	Couldn’t get “going”	–

“Reverse scored” refers to a method used in survey and psychological testing where certain questions are scored in the opposite direction to most other items on the scale. CESD-10, 10-item Center for Epidemiologic Studies Depression Scale.

#### 2.2.2 Primary independent variables

Primary independent variables included GS, sleep duration, and comorbid chronic diseases (CCDs). GS was measured using a handgrip dynamometer, which recorded the GS of the dominant hand in kilograms (kg). Sleep duration was assessed based on the number of hours (h) of nighttime sleep per day. Comorbidities were quantified by the number of chronic diseases reported, including hypertension, diabetes, and heart disease.

#### 2.2.3 Covariates

Covariates included gender, age, body mass index (BMI), lifestyle factors, and social activity participation. Lifestyle factors were detailed as smoking status (categorized as current smoker, former smoker, and never smoker), alcohol consumption (categorized as current drinker, former drinker, and never drinker), and physical activity intensity (classified into none, light, moderate, and vigorous).

### 2.3 Statistical analysis

#### 2.3.1 Descriptive analysis

Data were processed using SPSS (version 26.0). Continuous variables were presented as mean ± standard deviation or median (interquartile ranges, P25, P75), and categorical variables were summarized using frequencies and percentages. Differences in baseline characteristics between groups with and without depressive symptoms were compared using *t*-tests, Mann–Whitney U tests, or chi-square tests, as appropriate. Additionally, any missing data in the dataset were addressed through multiple imputations using the “mice” package.

#### 2.3.2 Subgroup analysis

Subgroup analyses were conducted to assess the influence of covariates, including gender, age, BMI, alcohol consumption, smoking status, social activity participation, and physical activity intensity, on the study outcomes. Forest plots were generated using R software (version 4.3.3) and the forestplot package to visually present the analysis results. Each stratified analysis was adjusted for factors such as gender, age (as a continuous variable), BMI (as a continuous variable), smoking status, alcohol consumption, social activity participation, and physical activity intensity. Binary logistic regression models were employed to calculate odds ratios (ORs) to quantify the strength of the association between each factor and the risk of depressive symptoms.

#### 2.3.3 Correlation analysis

Correlation analysis was conducted using R software (version 4.3.3) with the “corrplot” package to create heatmaps. The heatmaps illustrated the strength of correlations between nine specific depressive symptoms and variables such as GS, CCDs, and sleep duration.

#### 2.3.4 Network estimation and visualization

Network estimation was performed using the “bootnet” package in R (Epskamp et al., 2018), and visualization was achieved using the “qgraph” package (Epskamp et al., 2012). The network analysis was based on the Gaussian graphical model (GGM) and regularized using the graphical LASSO with the extended Bayesian information criterion (Epskamp and Fried, 2018) to limit the identification of spurious edges or connections between nodes, resulting in a more parsimonious model. The accuracy of edge weights and the stability of centrality metrics were assessed through a bootstrap procedure with 2,500 resamples (Epskamp et al., 2018; Robinaugh et al., 2016). Edge weights were determined based on the magnitude of partial correlations between variables after controlling for all other variables in the network. The following criteria were used to identify core symptoms: (1) Strength centrality: measuring the absolute sum of edge weights connected to each node; (2) Betweenness centrality: quantifying how often a node lies on the shortest path between other nodes; (3) Closeness centrality: calculating the inverse of the sum of shortest paths to all other nodes; Nodes with high values across these centrality measures were considered core symptoms.

## 3 Results

### 3.1 Baseline characteristics of participants

A total of 1,427 patients with CLDs were included in this study, comprising 794 males (55.6%) and 633 females (44.4%). Based on CESD-10 scores, 568 patients (39.8%) were identified with depressive symptoms (score ≥ 10). Significant differences were observed between the depressed and non-depressed groups in terms of gender, GS, number of CCDs, alcohol consumption, smoking status, and sleep duration (*p <* 0.05). Detailed baseline characteristics are presented in Table 2.

**Table 2:** Baseline characteristics of participants with and without depressive symptoms.

Variable	Total (1,427)	Non-depressed (859)	Depressed (568)	*p*-value
Gender				< 0.001^a^
Male	794 (55.6%)	535 (67.4%)	259 (32.6%)	
Female	663 (44.4%)	324 (51.2%)	309 (48.8%)	
Age (years)	63 (56, 70)	62 (55, 70)	63 (56, 69)	0.546^b^
BMI(kg/m^2^)	22.8 (20.4, 25.6)	22.8 (20.4, 25.6)	22.8 (20.4, 25.6)	0.798^b^
Grip strength	30.9 ± 9.4	32.4 ± 9.2	28.7 ± 9.2	< 0.001^c^
Comorbidities	2 (1, 3)	2 (1, 3)	2 (1, 3)	< 0.001^b^
Alcohol consumption				0.005^a^
Current drinker	491 (34.4%)	324 (37.7%)	167 (29.4%)	
Former drinker	235 (16.5%)	132 (15.4%)	103 (18.1%)	
Never drinker	701 (49.1%)	403 (46.9%)	298 (52.5%)	
Smoking status				< 0.001^a^
Current smoker	442 (31%)	277 (32.2%)	165 (29%)	
Former smoker	337 (23.6%)	228 (26.5%)	109 (19.2%)	
Never smoker	648 (45.4%)	354 (41.2%)	294 (51.8%)	
Physical activity intensity				0.305^a^
None	144 (10.1%)	81 (9.4%)	63 (11.1%)	
Light	348 (24.4%)	212 (24.7%)	136 (23.9%)	
Moderate	400 (28%)	254 (29.6)	146 (25.7%)	
Vigorous	535 (37.5%)	312 (36.3%)	223 (39.3%)	
Sleep duration	6 (5, 8)	6.5 (5, 8)	5 (4, 7)	< 0.001^b^
Social activity participation				0.426^a^
Yes	732 (51.3%)	448 (52.2)	284 (50%)	
No	695 (48.7%)	411 (47.8%)	284 (50%)	

Data are presented as mean ± standard deviation, M (P25, P75) or *n* (%); BMI, body mass index.

a Chi-squared test.

b Mann–Whitney U test.

c Independent-samples *t*-test.

### 3.2 Associations of GS, sleep duration, and CCDs with depressive symptoms in patients with CLDs

Binary logistic regression analyses were conducted, with adjustments made for gender, age, BMI, smoking status, alcohol consumption, social activity participation, and physical activity intensity. The findings uncovered significant inverse associations of GS (OR = 0.964, *p <* 0.001) and sleep duration (OR = 0.808, *p <* 0.001) with depressive symptoms. Conversely, a notable positive correlation was observed between the number of CCDs (OR = 1.189, *p <* 0.001) and depressive symptoms.

#### 3.2.1 Association between GS and depressive symptoms in patients with CLDs

**Figure 2:**
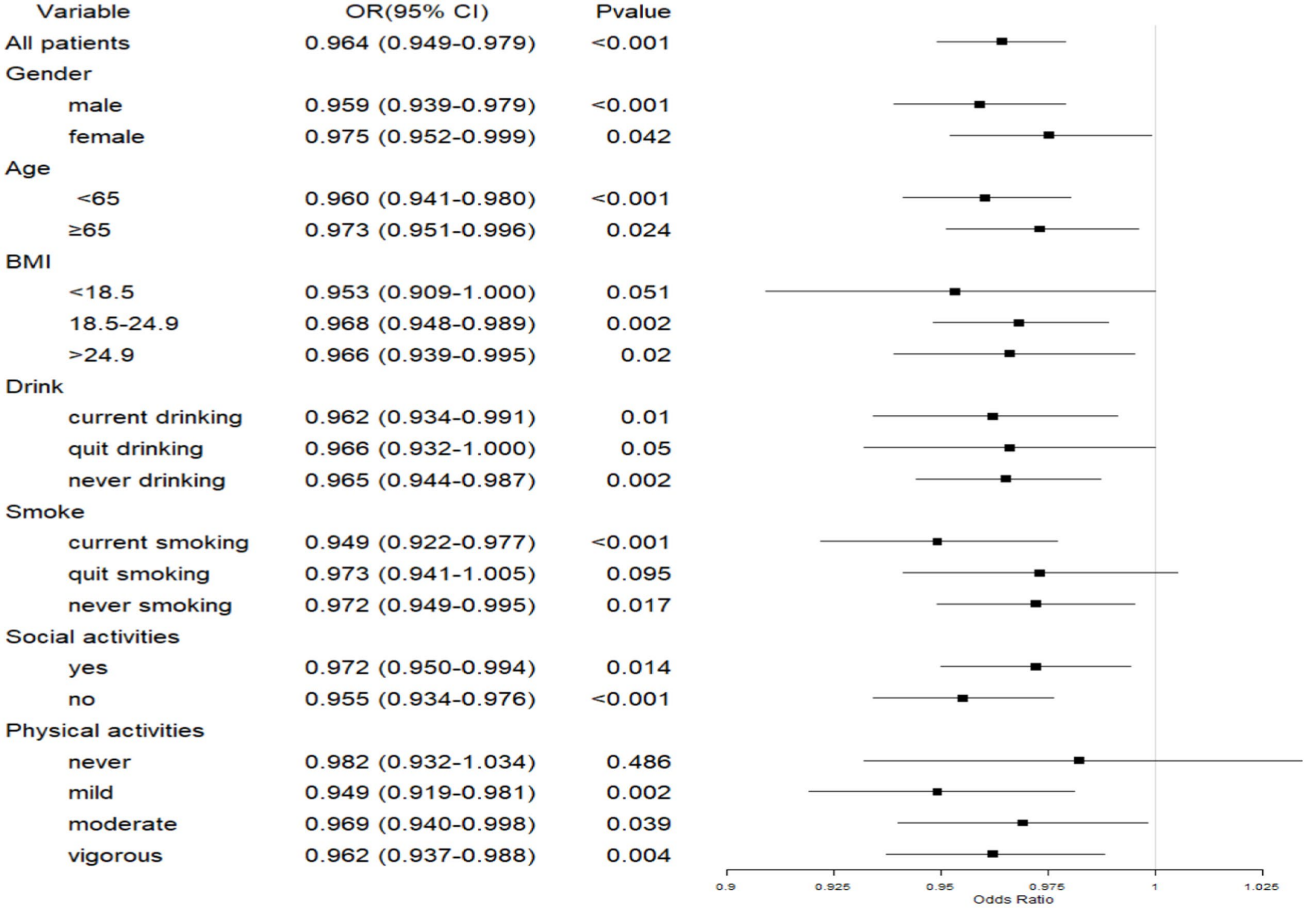
Forest plot of the association between GS and depressive symptoms in patients with CLDs. CLD, chronic lung disease; GS, grip strength; BMI, body mass index.

The subgroup analysis found a negative correlation between GS and depressive symptoms in both men and women, as well as across age groups (*p <* 0.05). This correlation was significant in social activity participants (*p <* 0.05) and non-participants (*p <* 0.001), individuals with normal to overweight BMI (8.5–4.9 kg/m^2^, *p <* 0.05), and those with obesity (BMI > 24.9 kg/m^2^, *p <* 0.05), but not in underweight individuals (BMI < 18.5). This suggests that individuals with normal or elevated BMI may be more likely to experience the mental health benefits associated with increased physical strength, while those underweight may have different underlying factors influencing their depressive symptoms that are not as strongly linked to physical strength. Among alcohol consumers, the correlation was significant in current drinkers (*p <* 0.05) and never drinkers (*p <* 0.05), but not in quit drinkers. For smokers, the correlation was strongest in current smokers (*p <* 0.001) and significant in never smokers (*p <* 0.05), but not in quit smokers. Physical activity levels were significantly associated with GS and depressive symptoms, with negative correlations observed in those engaging in mild, moderate, and vigorous exercise(OR = 0.949–0.969, p < 0.05). The lack of a significant association in individuals who did not engage in physical activity underscores the importance of regular exercise in maintaining both physical and mental health (Figure 2).

#### 3.2.2 Association between sleep duration and depressive symptoms in patients with CLDs

**Figure 3:**
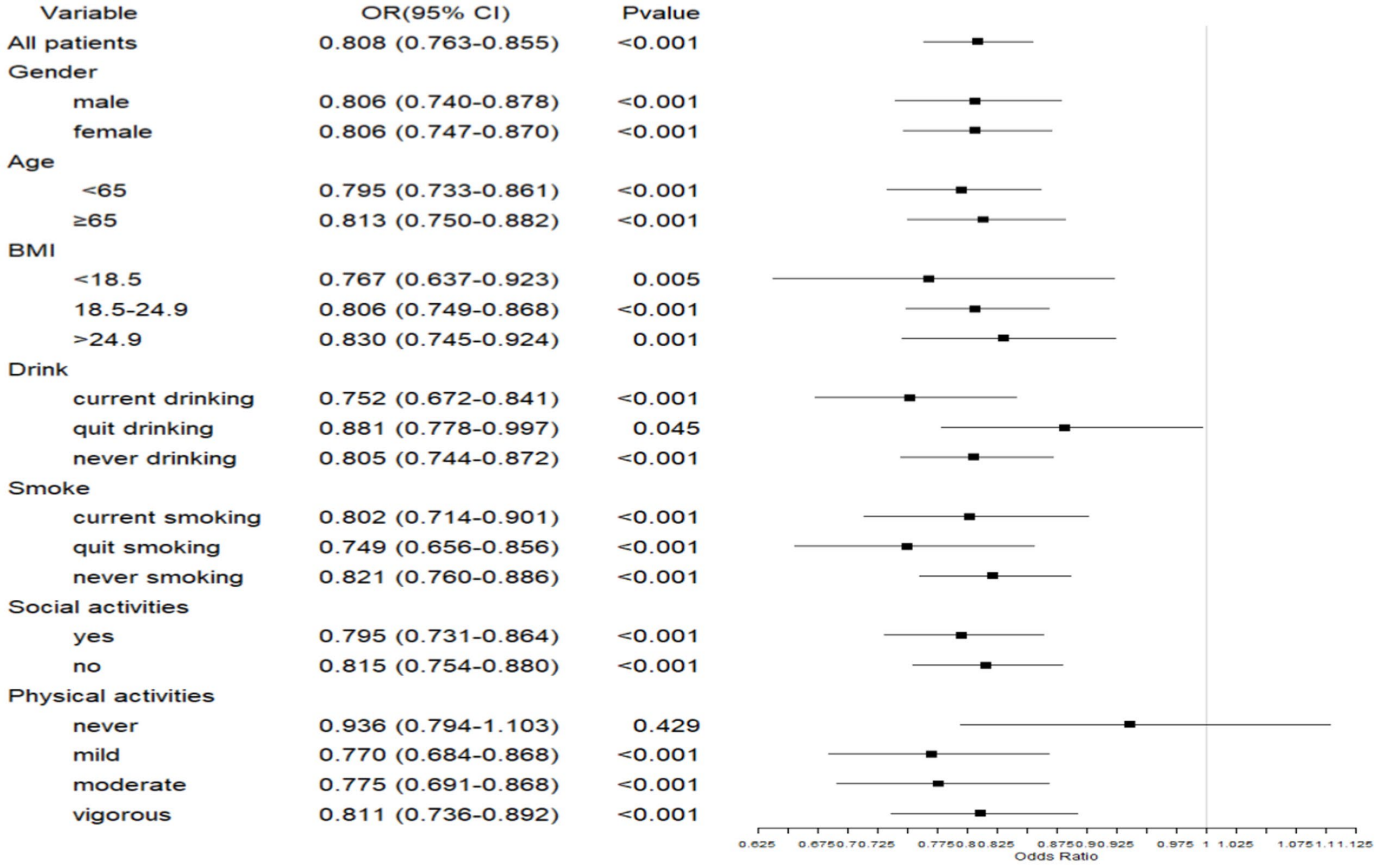
Forest plot of the association between sleep duration and depressive symptoms in patients with CLDs. CLD, chronic lung disease; BMI, body mass index.

Sleep duration was generally found to be significantly negatively associated with depressive symptoms across various subgroups, including gender, age, BMI, drinking and smoking status, social activity participation, and physical activity (*p* < 0.05). while those without physical activity had no significant association (*p* > 0.05) (Figure 3). These findings highlight the protective role of adequate sleep in mental health among individuals with CLD.

#### 3.2.3 Association between CCDs and depressive symptoms in patients with CLDs

**Figure 4:**
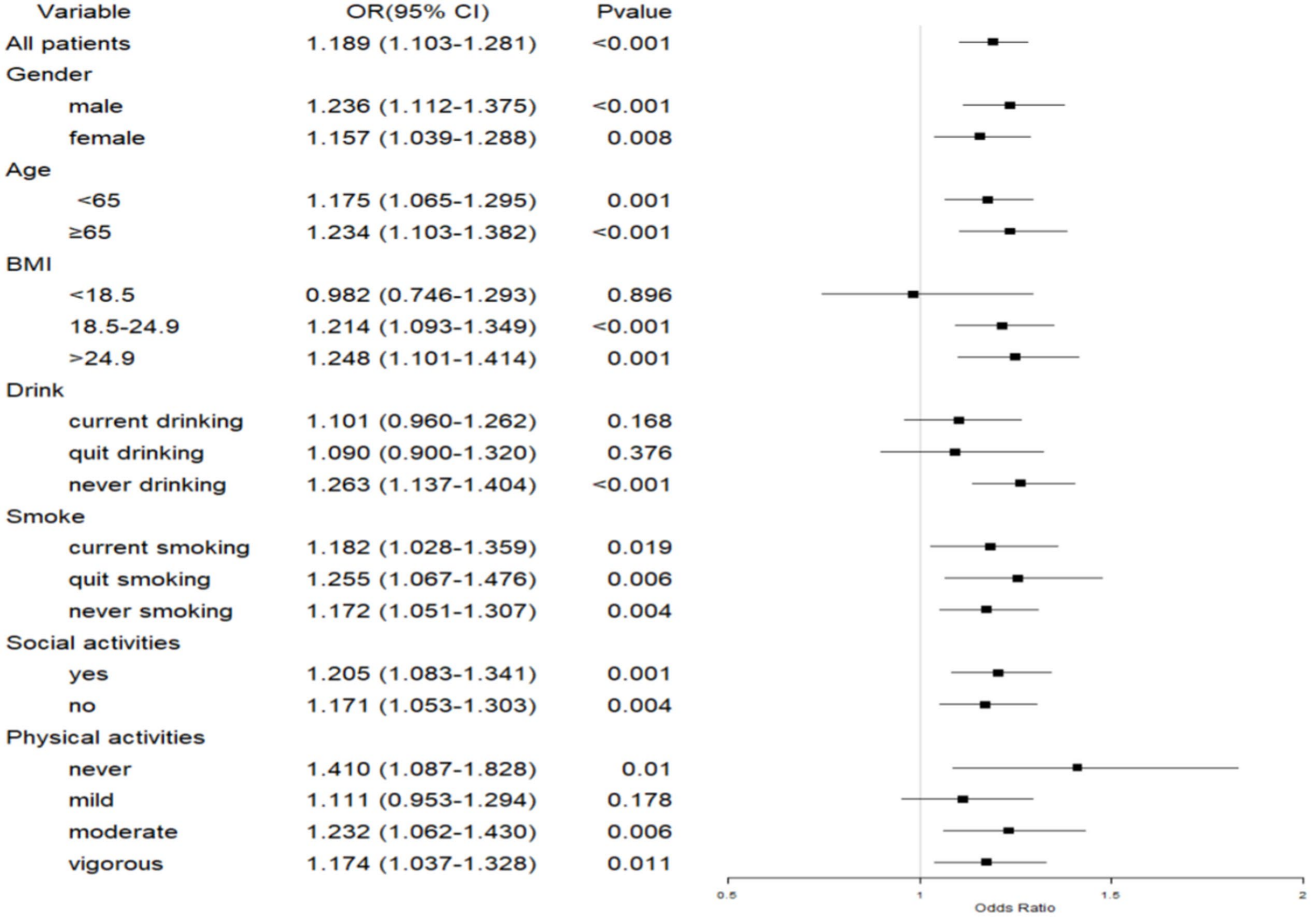
Forest plot of the association between CCDs and depressive symptoms in patients with CLDs. CLD, chronic lung disease; CCD, comorbid chronic disease; BMI, body mass index.

In patients with CLDs, the presence of CCDs was positively associated with depressive symptoms across different genders, age groups, smoking statuses, and both social activity participants and non-participants (*p* < 0.05). Significant associations were observed among those with a BMI in the normal to overweight range (18.5–24.9 kg/m^2^, OR = 1.214) and those with obesity (BMI > 24.9 kg/m^2^, OR = 1.248), while no significant association was found for underweight individuals (*p* > 0.05). Never drinkers showed a significant association with depressive symptoms (OR = 1.263, *p* < 0.001), while no association was found for current and quit drinkers. Additionally, non-exercisers and those engaging in moderate to vigorous physical activity showed significant associations (*p* < 0.05), whereas mild physical activity was not significantly associated (Figure 4).

### 3.3 Correlation analysis results using heatmaps

**Figure 5:**
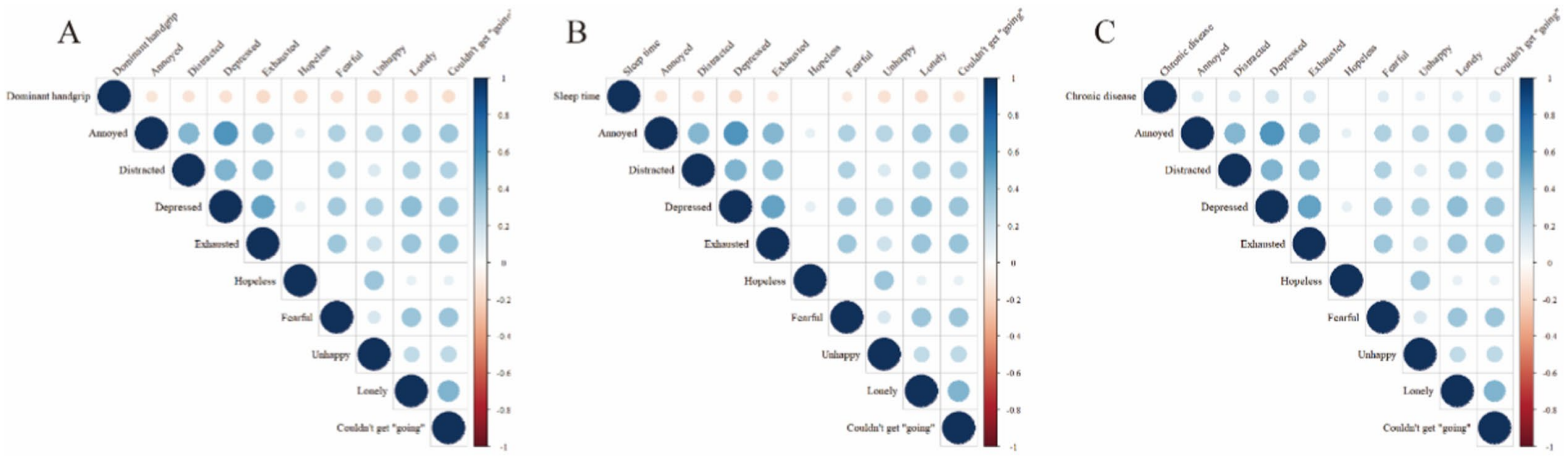
Correlations of GS, sleep duration, and CCDs with nine depressive symptoms. **(A)** Correlation of GS with nine depressive symptoms; **(B)** correlation of sleep duration with nine depressive symptoms; **(C)** correlation of CCDs with nine depressive symptoms. CLD, chronic lung disease; GS, grip strength; CCD, comorbid chronic disease.

The heatmap analysis unveiled a negative correlation between GS and all nine depressive symptom indicators (Figure 5A), highlighting that individuals with stronger GS exhibited milder depressive symptoms. In terms of sleep duration, no significant correlation was found with the symptom of hopelessness; however, negative correlations were evident with the other eight depressive symptoms (Figure 5B). This pattern underscored the potential association of longer sleep duration with fewer depressive symptoms. Furthermore, individuals with CCDs did not show a significant correlation with the hopelessness symptom, but positive correlations were witnessed with the other eight depressive symptoms (Figure 5C). This finding suggested that individuals with CCDs might be more susceptible to experiencing these depressive symptoms.

### 3.4 Network construction and centrality indicators in patients with CLDs

#### 3.4.1 Network analysis of depressive symptoms in patients with CLDs

**Figure 6:**
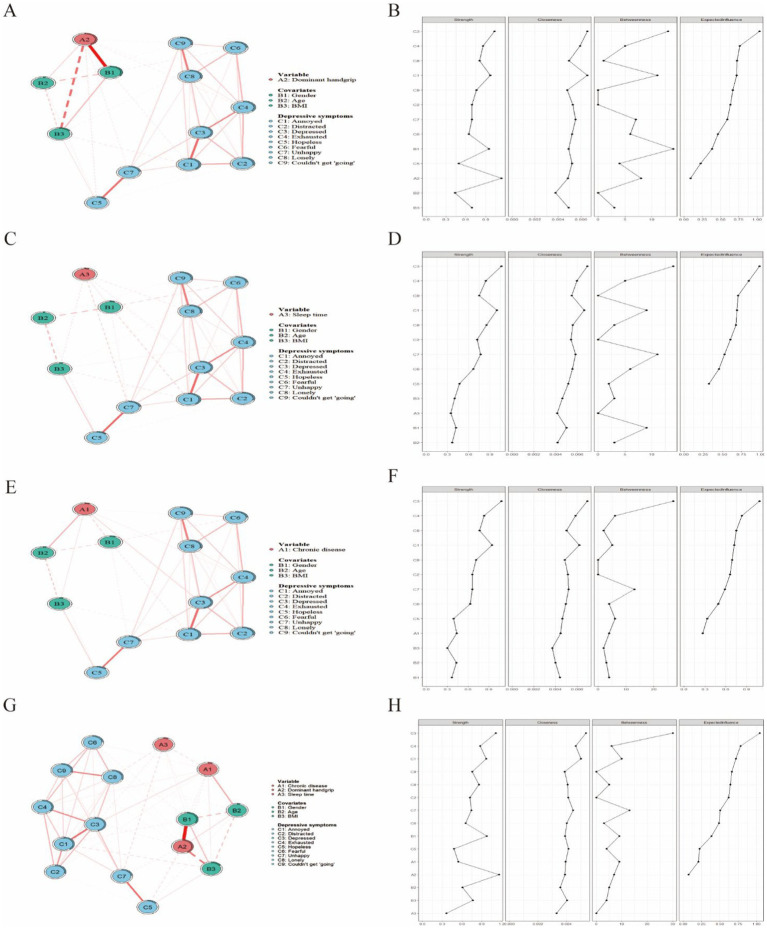
Network analysis of GS, sleep duration, CCDs, and depressive symptoms in patients with CLDs. **(A)** Network structure of GS and depressive symptoms; **(B)** centrality metrics for GS and depressive symptoms; **(C)** network structure of sleep duration and depressive symptoms; **(D)** centrality metrics for sleep duration and depressive symptoms; **(E)** network structure of CCDs and depressive symptoms; **(F)** centrality metrics for CCDs and depressive symptoms; **(G)** network structure of GS, sleep duration, CCDs and depressive symptoms; **(H)** centrality metrics for GS, sleep duration, CCDs and depressive symptoms. In the network analysis diagrams (panels **A,C,E,G**), each variable is represented as a node, and the variables are connected by edges. The thickness of the edges indicates the strength of the correlations. In the centrality metrics (panels **B,D,F,H**), the X-axis represents standardized z-scores, and the Y-axis represents symptom items. CLD, chronic lung disease; GS, grip strength; CCD, comorbid chronic disease.

Network analysis, with age, gender, and BMI as covariates, was utilized to illustrate the complex associations of GS, sleep duration, and CCDs with the nine depressive symptoms. As depicted in Figures 6A–H, all three network diagrams displayed similar connectivity patterns. The symptom labeled “depressed” (C3) was identified as the core symptom of depression in the network with the highest levels of predictability and mediating effects. it exhibited the highest strength centrality (2.14), betweenness centrality (1.89), and closeness centrality (1.76). Secondary core symptoms (“annoyed,” “exhausted,” and “lonely”) showed moderately high centrality values but were less central than “depressed.” The network structure demonstrated good stability with: CS-coefficient for strength centrality = 0.75, CS-coefficient for betweenness centrality = 0.67, CS-coefficient for closeness centrality = 0.71, These values exceed the recommended threshold of 0.5, indicating reliable centrality estimates.

Particularly, “annoyed” (C1) and “exhausted” (C4) were closely linked to “depressed” (C3), which formed the central symptom cluster within the network. This clustering indicates that patients with CLDs manifesting primary “depressed” symptoms are predisposed to a range of interconnected emotional states, such as “annoyed” and “exhausted” symptoms, along with further associated symptoms such as “distracted” (C2), “fearful” (C6), and “lonely” (C9).

The analysis of edge weights revealed robust links between GS and “annoyed” (C1), “exhausted” (C4), “hopeless” (C5), and “unhappy” (C7). Sleep duration showed close connections with “depressed” (C3), “lonely” (C8), and “unhappy” (C7). Furthermore, the number of CCDs was robustly linked with the symptoms “depressed” (C3) and “exhausted” (C4).

The centrality metrics in the GS network revealed that the symptom “annoyed” (C1) was closely associated with GS and served as a secondary core symptom. It ranked second in both closeness and betweenness centrality, suggesting that “annoyed” might confer a crucial mediating role between GS and other depressive symptoms. Similarly, in the networks concerning sleep duration and CCDs, the symptom “depressed” (C3) exhibited this significant mediating role. These findings pointed to the potential of “annoyed” and “depressed” as key mediators in the network for targeted therapeutic strategies against depressive symptoms.

#### 3.4.2 Stability analysis of depressive symptom networks

**Figure 7:**
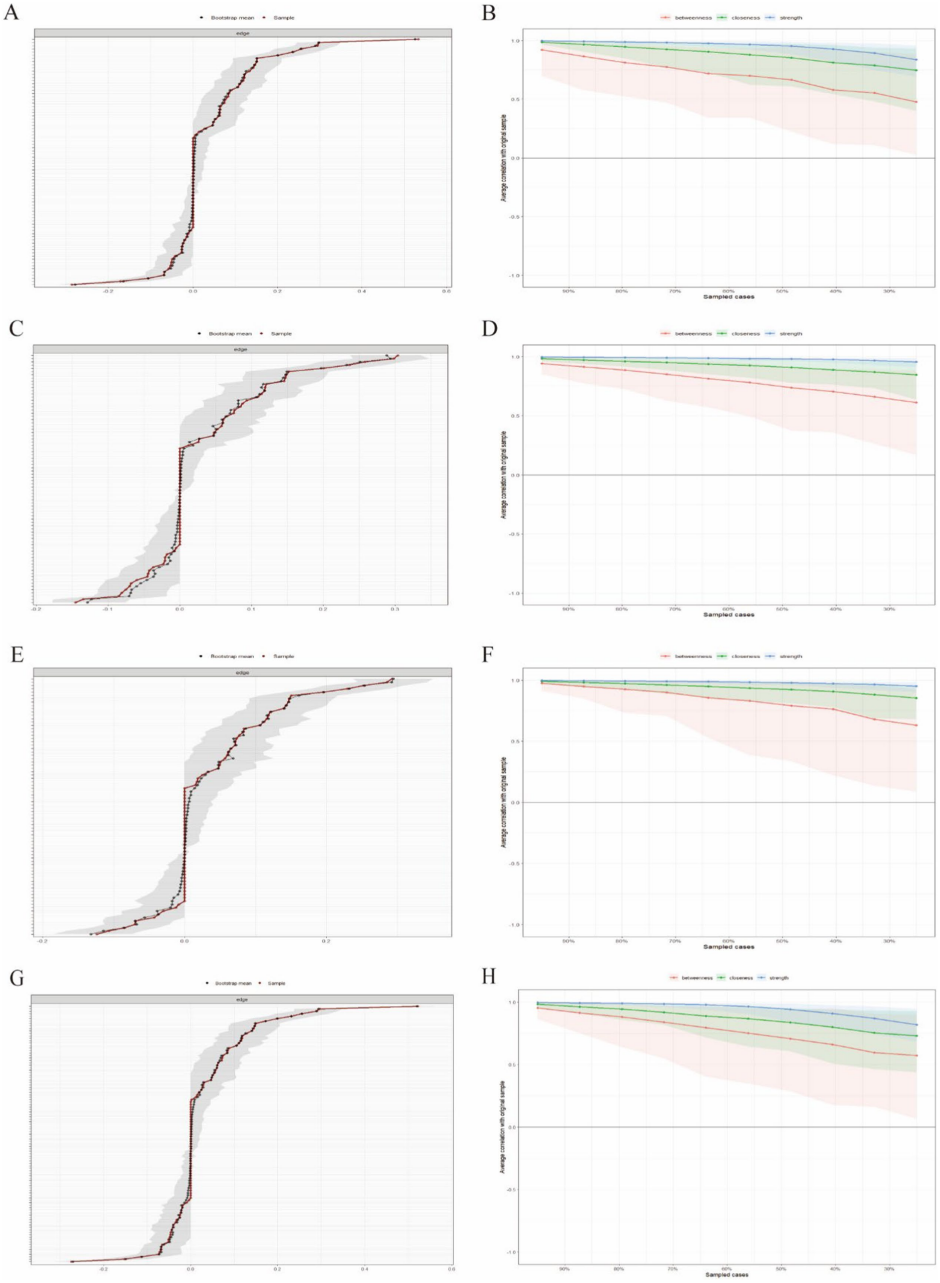
Network stability analysis. **(A,B)** Stability analysis of the network involving GS; **(C,D)** stability analysis of the network involving sleep duration; **(E,F)** stability analysis of the network involving CCDs. **(G,H)** stability analysis of the network involving the union of GS, sleep duration and CCDs; In panels **A,C,E,G**, the stability of edge weights is displayed. The black line represents the mean derived from the bootstrap method, the red line represents the mean estimated from the original sample, and the gray area represents the confidence intervals corresponding to both methods. Greater overlap of the lines and a smaller gray area reflect more stable results. In panels **B,D,F,H**, the stability of centrality metrics is tested. The test examines whether the order of centrality metrics remains consistent as the sample size or number of nodes decreases. The quantification metric is the correlation coefficient of stability, which represents the maximum acceptable reduction in sample size. The minimum acceptable CS is 0.25. CCD, comorbid chronic disease.

Bootstrapping, with 2,500 samples, was employed to examine the accuracy and stability of the network (Figures 7A–H). The evaluation of network stability indices of the centrality metrics uncovered excellent stability of strength across all three network structures, indicating that 95% of the sample could be omitted while still maintaining a correlation coefficient of 0.7, with the network structure remaining consistent with the original configuration (Figures 7B,D,F,G). Furthermore, the consistency of edge weights was verified by their overlapping 95% confidence intervals, as illustrated in Figures 7A,C,E,H. These results underscore the high robustness of the network in patients with CLDs.

## 4 Discussion

This study, based on data from the CHARLS, utilized binary regression analysis, subgroup analysis, correlation analysis, and network analysis to comprehensively investigate the associations and underlying mechanisms of GS, sleep duration, and CCDs with depressive symptoms in patients with CLDs. The findings illuminated complex relational networks between these factors and depressive symptoms, offering new perspectives for understanding and intervening in depressive symptoms among patients with CLDs. The 39.8% prevalence of depressive symptoms underscores the severity of depression in patients with CLDs and highlights the critical need for mental health support within this group.

Our findings confirmed a significant negative correlation between GS and depressive symptoms, corroborating previous research findings. For instance, Gu et al. (2021) uncovered that lower GS was associated with a higher risk of depression in a large-scale cohort study of the general adult population in China. Our research extends this finding to patients with CLDs, and highlights that this association is particularly pronounced in various subgroups such as both men and women and different age groups. The correlation was also significant regardless of social activity participation, indicating its broad relevance in the context of CLDs. These observations propose that GS could serve as a straightforward and effective indicator for assessing and preventing depressive symptoms in patients with CLDs. Reduced GS, indicative of overall muscle strength, may reflect a decline in physical function in patients with CLDs, which may adversely affect their mental health (Sepúlveda-Loyola et al., 2020). The deterioration of physical function is often accompanied by limited physical activity, which in turn increases social isolation and feelings of helplessness, all of which are known risk factors for depression. Additionally, the decline in muscle strength in patients with chronic lung disease may be associated with chronic low-grade inflammation, which is considered an important biological mechanism of depression (Heresco-Levy et al., 2024). At the same time, fatigue is a common symptom in patients with chronic lung disease, and low grip strength may be associated with increased fatigue. Fatigue restricts participation in daily activities, leading individuals to feel unable to cope with everyday challenges, thus increasing the risk of depression. Furthermore, our research validated the negative correlation between sleep duration and depressive symptoms (Gebara et al., 2018; Fang et al., 2019). This relationship was more prominent in subgroups of younger individuals, underweight individuals, current drinkers, and those engaged in moderate physical activities. These findings highlight the critical role of sleep quality improvement in both the preventive and therapeutic management of depressive symptoms among patients with CLDs. Sleep disorders may exacerbate depressive symptoms in patients with CLDs by affecting the neuroendocrine system and immune function. Importantly, our study underscores the relevance of sleep duration as a quantifiable measure and a practical intervention target for clinical settings. Additionally, a positive correlation exists between the number of CCDs and depressive symptoms, with this association being more significant among older males, never drinkers, and those with low physical activity intensity. This finding suggests that the coexistence of multiple chronic conditions may augment the psychological burden on patients with CLDs, thereby increasing their susceptibility to depression. These findings are consistent with prior meta-analyses, which highlighted the detrimental impact of COPD coexisting with multiple chronic diseases on mental health. Our study further quantified this relationship and identified specific high-risk subgroups, offering enhanced precision in guiding clinical practice to mitigate these risks.

The network analysis results further unveiled the complex relationships between depressive symptoms and factors such as GS, sleep duration, and CCDs. The symptom “depressed,” serving as a core symptom, is extensively connected with other symptoms and factors, indicating its potential role as a key node driving the entire symptom network. Moreover, the robust associations between various factors and specific depressive symptoms (such as GS with “exhausted” and “unhappy,” and sleep duration with “lonely”) suggest potential directions for targeted interventions. These observations are consistent with the depressive symptom network theory proposed by Fried et al. (2016), which underscores the critical need to comprehend the interactions among symptoms. Our study extended this theoretical framework to patients with CLDs and provided more detailed and specific insights into depression in this specific patient population.

These findings carry important implications for clinical management and public health strategies in patients with CLDs. The assessment of GS emerges as a crucial aspect. As a straightforward, rapid, and cost-effective diagnostic measure, GS can be incorporated into the routine assessments of patients with CLDs. Its utility extends beyond evaluating physical function and also acts as a predictive indicator for depression risk. Consequently, clinicians are advised to consider enhanced psychological health surveillance and proactive interventions for individuals exhibiting diminished GS. This recommendation resonates with the suggestions provided by Celis-Morales et al. (2018), who also underscored the value of the incorporation of GS evaluations into routine health assessments. The study also highlights the pivotal role of sleep management in maintaining the mental health of patients with CLDs. Healthcare providers are advised to intensify their focus on sleep disturbances, facilitate education on sleep hygiene, and implement behavioral or pharmacological treatments as needed to improve sleep quality in this specific patient population. Enhanced sleep management is particularly crucial for younger patients, underweight individuals, and individuals who regularly consume alcohol, as they may experience more significant mental health benefits from improved sleep management. Correspondingly, this finding aligns with the research conducted by Omachi et al. (2012), which highlighted the importance of improving sleep quality in patients with COPD. Moreover, integrated management for patients with CLDs and multiple chronic conditions is another essential aspect highlighted by the study. An integrated management strategy should include the coordination of multidisciplinary teams to develop holistic treatment plans that cater to both the physical and psychological dimensions of patient care. This is particularly important for older males and individuals with low physical activity intensity, who may benefit from more proactive mental health interventions. This recommendation is consistent with the integrated management model for patients with COPD proposed by Yohannes and Alexopoulos (2014). Lastly, network analysis has provided fresh insights into network-based precision interventions in clinical practice. The symptom “depressed” as the core symptom in the network suggests the necessity for clinicians to focus intensely on patients’ depressive states and recognize their potential role in initiating or intensifying other health issues. Additionally, targeted interventions can be designed based on the robust associations between various factors and specific depressive symptoms (such as GS with “exhausted” and “unhappy” and sleep duration with “lonely”). For example, in patients with diminished GS, an integrated approach involving both physical rehabilitation and psychological support could be employed to simultaneously alleviate fatigue and improve emotional well-being.

Despite the promising insights this study provides, it is important to recognize its limitations. As a cross-sectional study, this study cannot ascertain causal relationships between GS, sleep duration, CCDs, and depressive symptoms, which may have a bidirectional pattern. For instance, depressive symptoms might impair sleep quality, which in turn could exacerbate depressive symptoms. Furthermore, despite the adjustment for numerous potential confounders, unmeasured confounding variables could still affect the results. Factors such as social support, economic status, and the severity of the disease may influence the risk of depression in patients with CLDs. Future research should aim to overcome these limitations by employing longitudinal cohort studies to investigate the long-term effects of GS, sleep quality, and CCDs on depressive symptoms in patients with CLDs. The development and meticulous evaluation of intervention strategies derived from this study’s outcomes, such as integrated programs combining GS training and psychological support, could substantially aid in the prevention and amelioration of depression. It is imperative to delineate the biological underpinnings that link GS, sleep duration, and CCDs with depressive symptoms through inflammatory responses, oxidative stress, or neuroendocrine pathways. Advanced network analysis techniques, such as time-series network analysis, could shed light on the dynamic interactions between depressive symptoms and related influencing factors. Multicenter and cross-cultural studies are necessary to validate the applicability of these findings in diverse populations and healthcare systems, ensuring their relevance and utility across different contexts.

Therefore, this study unveiled the intricate associations of GS, sleep duration, and CCDs with depressive symptoms in patients with CLDs. These findings offer new insights and potential intervention targets to manage the psychological health of this vulnerable population. Future research should focus on clarifying the causal mechanisms underlying these relationships and on developing evidence-based, individualized intervention strategies to enhance mental health and overall quality of life for patients with CLDs.

## 5 Conclusion

This research contributes to the understanding of the interplay between physical health and psychological well-being in patients with CLDs and highlights significant implications for clinical practice and patient care. The identification of GS and sleep duration as potential modifiable risk factors suggests avenues for clinical intervention that could mitigate depressive symptoms in patients with CLDs. Future research should focus on longitudinal studies and diverse populations to validate these findings and develop effective, evidence-based interventions.

## Data Availability

The original contributions presented in the study are included in the article/supplementary material, further inquiries can be directed to the corresponding author.
